# Differential Susceptibility of *Mycoplasma* and *Ureaplasma* Species to Compound-Enhanced Copper Toxicity

**DOI:** 10.3389/fmicb.2019.01720

**Published:** 2019-07-30

**Authors:** Arthur H. Totten, Cameron L. Crawford, Alex G. Dalecki, Li Xiao, Frank Wolschendorf, Thomas P. Atkinson

**Affiliations:** ^1^Department of Pediatrics, The University of Alabama at Birmingham, Birmingham, AL, United States; ^2^Department of Medicine, The University of Alabama at Birmingham, Birmingham, AL, United States

**Keywords:** mollicutes, copper, drug discovery, disulfiram, GTSM, neocuproine, *Mycoplasma*, *Ureaplasma*

## Abstract

**Rationale:**

Mycoplasmas represent important etiologic agents of many human diseases. Due to increasing antimicrobial resistance and slow rate of novel discovery, unconventional methods of drug discovery are necessary. Copper ions are utilized in host microbial killing, and bacteria must regulate intracellular Cu concentrations to avoid toxicity. We hypothesized that human mollicutes may have susceptibility to Cu-induced toxicity, and compounds that augment copper-dependent killing.

**Methods:**

*Mycoplasma pneumoniae* (Mpn), *Ureaplasma parvum* (Up), *Ureaplasma urealyticum* (Uu), and *Mycoplasma hominis* (Mh) were exposed to CuSO_4_ to determine minimal inhibitory concentrations (MICs). Once inhibitory concentrations had been determined, bacteria were treated with an FDA-approved drug disulfiram (DSF), glyoxal bis(4-methyl-3-thiosemicarbazone) (GTSM), and 2,9-dimethyl-1,10-phenanthroline (neocuproine), with or without Cu^2+^, to determine compound MICs.

**Results:**

*Ureaplasma* species and Mh were able to tolerate 30–60 μM CuSO_4_, while Mpn tolerated over 10-fold higher concentrations (>1 mM). GTSM inhibited growth of all four organisms, but was unaffected by Cu^2+^ addition. Inhibition by GTSM was reduced by addition of the cell-impermeant Cu chelator, bathocuproine disulfonate (BCS). Neocuproine exhibited Cu-dependent growth inhibition of all organisms. DSF exhibited Cu-dependent growth inhibition against Mh at low micromolar concentrations, and at intermediate concentrations for Mpn.

**Conclusion:**

MICs for CuSO_4_ differ widely among human mollicutes, with higher MICs for Mpn compared to Mh, Uu, and Up. DSF and Neocuproine exhibit Cu-dependent inhibition of mollicutes with copper concentrations between 25 and 50 μM. GTSM has copper-dependent anti-microbial activity at low levels of copper. Drug enhanced copper toxicity is a promising avenue for novel therapeutic development research with *Mycoplasma* and *Ureaplasma* species.

## Introduction

Mollicutes are a class of bacteria that lack a cell wall and are important etiologic agents of many human diseases. Global antibiotic resistance rates in mollicutes differ based on bacterial species, geographical location, and patient population, but rising antibiotic resistance is a concern for some species and antibiotic classes. The trends toward increasing antimicrobial resistance worldwide underscore the need for novel antimicrobial compound discovery. The importance of copper in macrophage-mediated microbial killing has suggested that copper-transporting compounds might prove useful as antimicrobial agents (White et al., 2009).

Screening compounds for copper-dependent killing activity has resulted in the identification of new compounds with copper-dependent antimicrobial activity against *Mycobacterium tuberculosis* and *Staphylococcus aureus* (Dalecki et al., 2016; Shah et al., 2016). Previous work has been described examining the effect of antimicrobial compounds on copper-induced stress in *Mycoplasma gallisepticum* (Smit et al., 1982; Gaisser et al., 1987a,b; de Zwart et al., 1991), but to date studies in this area have not been conducted with human mycoplasmas or ureaplasmas. In the current study, we sought to determine whether compounds with Cu-binding activity exhibit antimicrobial activity against human mollicutes.

## Materials and Methods

### Drugs and Compounds

Copper sulfate, iron sulfate heptahydrate (FeSO_4_), zinc sulfate heptahydrate (ZnSO_4_), and manganese dichloride (MnCl_2_) were purchased from Sigma-Aldrich, dissolved in water as 100 mM stock solutions and stored at 4°C. The FeSO_4_ solution was always prepared fresh prior to use. Bathocuproinedisulfonic acid was purchased from Fisher Scientific and stored as a 100 mM stock solution in water at −80°C. Stocks of test compounds were prepared in DMSO and stored at −80°C as follows: Neo (Sigma-Aldrich) 10 mM, DSF (Sigma-Aldrich) 40 mM, bathocuproine (Sigma-Aldrich) 1 mM, and GTSM 10 mM. GTSM was a kind gift from Dr. Stefan Bossmann, who synthesized the compound in his laboratory at Kansas State University following published protocols (Haeili et al., 2014).

### Bacterial Strains and Culture

Bacterial strains for each species were utilized for *in vitro* analyses throughout the course of this work. Clinical isolates were the generous gift of Dr. Ken B. Waites at the University of Alabama at Birmingham Diagnostic Mycoplasma Laboratory. For *Mycoplasma pneumoniae*, strains M129 (ATCC 29342), FH (ATCC 15531), UAB PO1 (Clinical isolate), and 54524 (Clinical isolate, strain also known as UAB JZY) were used. *Ureaplasma parvum* and *Ureaplasma urealyticum* isolates were: Up3 (serovar 3, ATCC 700970), Uu10 (serovar 10, ATCC 33699), and Uu9 (serovar 9, ATCC 33175) (Xiao et al., 2014). *Mycoplasma hominis* isolates included the type strain PG21 (ATCC 23114), and 55391 (clinical isolate).

Bacteria were cultured in SP4 or 10B broth, and SP4 or A8 agar prepared by the UAB Diagnostic Mycoplasma Laboratory, as described previously (Waites et al., 2012b; Xiao et al., 2014; Totten et al., 2017). In brief, bacterial isolates were thawed from frozen stocks at known concentrations prior to assay conditions, and then diluted as per laboratory standards for human mollicutes MIC assays (Waites et al., 2012a). Bacterial CFU/mL concentrations ranged from 10^4^ to 10^5^ for tested species to ensure accurate susceptibility testing. Growth of all mollicutes species was carried out prepared as previously described. Mpn strains were grown in SP4 broth at 37°C for 5–7 days and examined for yellow-orange color change. Mh isolates were additionally grown in SP4 broth, sealed with adhesive plate sealers and incubated at 37°C for 1–3 days and examined for color change. *Ureaplasma* spp. were cultured in 10B broth, sealed with adhesive plate sealers, and grown at 37°C for 1–2 days prior to media color change. CFU/mL concentrations were additionally examined for each plate to ensure accurate CFU range on solid media (SP4 agar, or A8 agar) as described previously (Xiao et al., 2014; Totten et al., 2017). Growth index measurements (Feng et al., 2018) were carried out on a microplate reader (Cytation 3, Biotek) by calculating the absorption ratio as follows for each species: Mpn (A430/A560) and Mh/*Ureaplasma* spp. (A560/A430).

### Metal Content Analysis

A milliliter of SP4 and 10B was dried overnight in a 65°C oven. The dried pellet was suspended in 500 μL trace metal grade nitric acid (∼70%, Fisher Scientific) and incubated at 65°C for 6 h. The samples were further diluted to 2% nitric acid in LC/MS grade water (Fisher Scientific) prior to analysis. The media was analyzed for the content of six metals by ICP-MS (Agilent) (Cu, Ca, Fe, Mn, Mg, and Zn) and reported as micromolar concentrations. Metal content was determined by comparison to a standard curve (Millipore) with the detection limit of 275 nM copper. The machine was calibrated using Lithium, Scandium, Germanium, and Indium as internal standards (High Purity Standards). Samples were analyzed in triplicate, and error bars represent standard deviations. Data were analyzed using Agilent’s offline data analysis program.

### Cu and Compound Microdilutions

For metal toxicity studies, CuSO_4_ or other transition metals were serially diluted 1:2 from 1 mM to 1.9 μM in SP4 or 10B broth. Bacteria were added to respective wells and incubated as outlined earlier. The assay working concentration for exogenous Cu for each organism was lower than that showing inhibition of bacterial growth *in vitro* (Table 1).

**TABLE 1:** Elemental analysis of media types for growth of mollicutes *in vitro* by ICP-MS.

**Element**	**10B broth (μM ± SD)**	**SP4 broth (μM ± SD)**	**Sig. different? (*P* = *x*)**
Ca	8.14 (±0.03)	14.64 (±0.84)	0.0002
Cu	0.66 (±0.13)	0.21 (±0.02)^*^	0.0042
Fe	1.95 (±0.05)	1.50 (±0.07)	0.0010
Mg	32.7 (±0.39)	34.6 (±1.36)	0.0846
Mn	0.10 (±0.001)^*^	0.068 (±0.003)^*^	0.0002
Zn	1.65 (±0.02)	2.34 (±0.10)	0.0003

^*^Denotes that measurement of element was below the limit of detection during ICP-MS analysis.

For compound or drug MICs, broth medium was diluted with or without transition metals at predetermined working concentrations tolerated by each bacterial strain. Compounds for MIC testing were added in serial twofold dilutions up to 10 μM. For MICs in media containing bathocuproine, the compound was diluted to 500 μM final concentration in medium with bacteria for 1 h at 37°C prior to addition to MIC plates. Bacteria were then added to respective wells and their rates of growth measured over time as described above.

### Statistical Analysis

Unless otherwise noted, sample means ± SD were utilized for comparison of statistical significance. When data were normally distributed, comparison of more than two experimental groups was performed utilizing One-way ANOVA with Bonferroni pair-wise post-tests or Tukey *ad hoc* post-test. Where appropriate, two-way ANOVAs were utilized for data groups with more than two independent variables. Log_10_ transformation was utilized to increase the likelihood of Gaussian distribution on specific data sets. Non-parametric data were analyzed utilizing the Mann–Whitney *U* test, with a Dunn’s *post hoc* test for pairwise comparisons. With data sets containing two experimental groups, unpaired *t*-tests were utilized for analysis. One-tailed tests were utilized to increase statistical significance where prior data had indicated directionality under two-tailed conditions. Data were graphed using Graphpad PRISM v. 8 (Graphpad Software, Inc.). Differences were considered statistically significant when *p* < 0.05. For significance values the following symbols were used: ^*^ for *p* < 0.05, ^∗∗^ for *p* < 0.01, ^∗∗∗^ for *p* < 0.001, and ^****^ for *p* < 0.0001.

## Results

### MIC for Copper Is Higher for Mpn Than for Urogenital Mollicutes

Prior to examination of susceptibilities of human mollicutes to antimicrobial compounds, elemental analysis was undertaken on samples of the broth media 10B and SP4, which were used to grow *Mycoplasma* and *Ureaplasma* spp. in the subsequent experiments. Sterile broth was subjected to elemental analysis by ICP-MS. Elemental analysis carried out for calcium, copper, iron, magnesium, manganese, and zinc showed significant differences between 10B and SP4 (Table 1), but importantly for the studies reported here, the copper concentration was less than 1 μM in both media. 10B had significantly more elemental Cu and Fe and lower amounts of Ca and Zn than SP4. Mn was below the limit of detection (LoD) in both media types, while only SP4 had levels of Cu below the LoD of the ICP-MS (Table 1).

**FIGURE 1:**
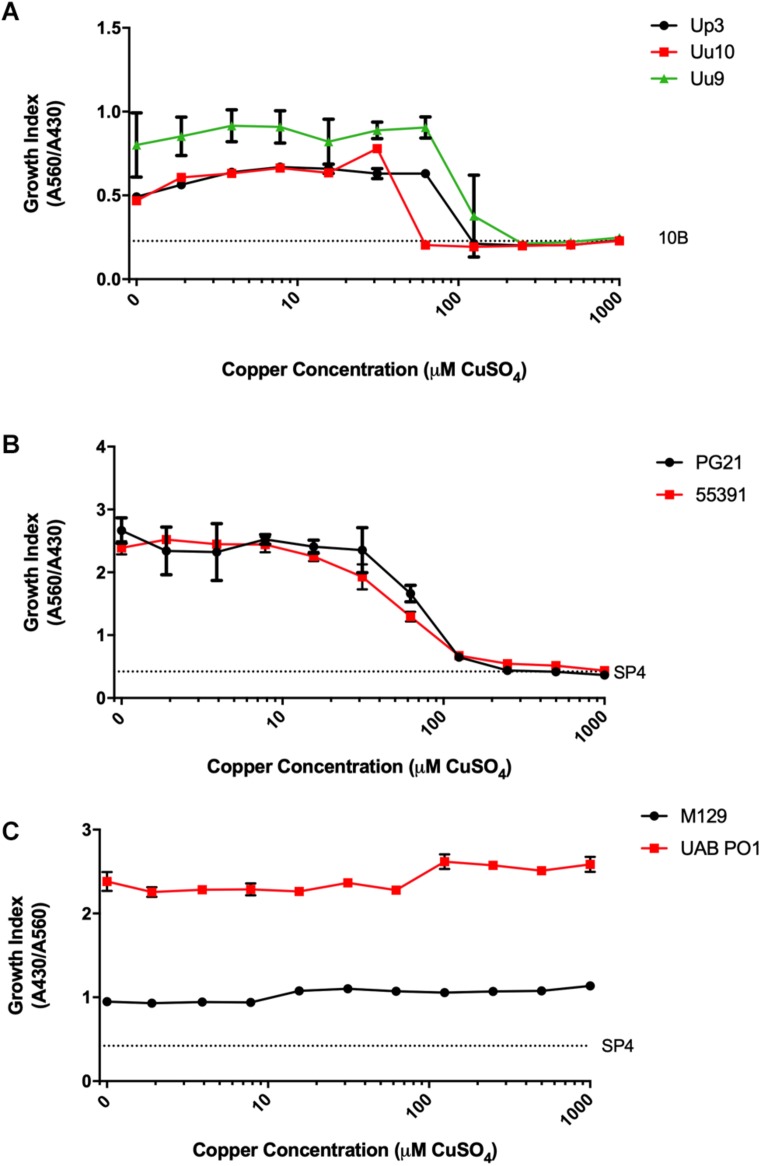
Differential susceptibility of Mollicutes to Cu stress *in vitro*. Growth of the indicated Mollicute species across three logs of Cu concentrations **(A)** *U. parvum* serovar 3, *U. urealyticum* serovar 9, and *U. urealyticum* serovar 10; **(B)** *M. hominis* strains PG21 and 55391; and **(C)** Mpn strains M129 and UAB PO1. Graphs depict mean ± SD (*n* = 3 replicates). Experiments were repeated 2–3 times at minimum.

Screening was carried out on Mpn, Mh, Up, and Uu isolates to determine the MICs for copper by adding serial dilutions of CuSO_4_ to broth cultures. *Ureaplasma* strains Up serovar 3 (Up3) and Uu serovar 9 (Uu9) were inhibited completely at concentrations above 60–120 μM Cu, but Uu serovar 10 (Uu10) was unable to tolerate levels greater than 30–60 μM (Figure 1A). MICs for Mh strains PG21 and UAB 55391 were comparable, with complete inhibition above 60–120 μM Cu (Figure 1B). Surprisingly, Mpn strains M129 and UAB PO1 (as well as strains FH and UAB JZY, not shown) were all able to tolerate concentrations up to 1 mM CuSO_4_ (Figure 1C). Due to acidification of the broth with addition of greater than 1 mM CuSO_4_, testing above this concentration could not be done using the chosen method. Total human serum copper levels average 17 μM (range 7–41), while estimated concentrations in the phagolysosome are predicted to be 10–20-fold (range 100–300 μM) higher than that found in plasma (Twomey et al., 2005; Wagner et al., 2005; White et al., 2009). Based on the MIC results with copper, further testing for an effect of selected compounds with copper was performed using 50 μM exogenous CuSO_4_ for all strains except Uu10, for which 25 μM was used.

### Cu-Binding Compound Neocuproine Exhibits Cu-Dependent Antimicrobial Activity Against Human Mollicutes

**FIGURE 2:**
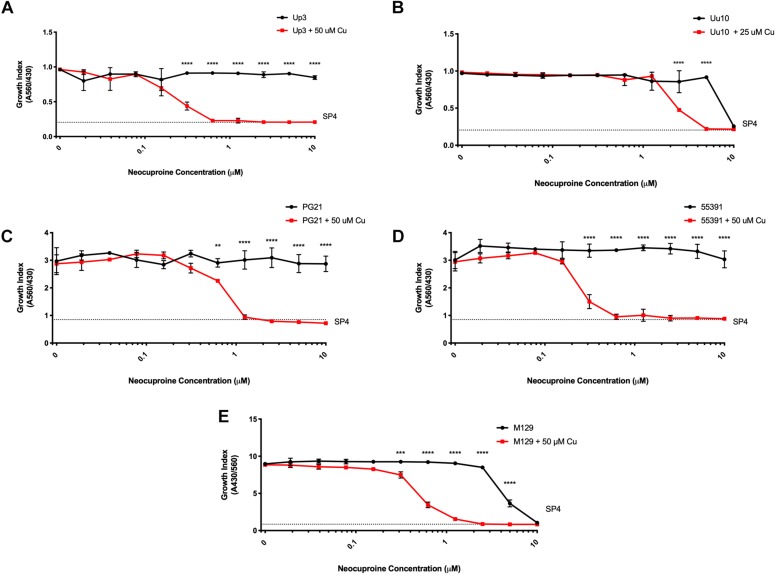
Neocuproine shows Cu-enhanced toxicity *in vitro* against *Ureaplasma* and *Mycoplasma* species. Representative growth indices of Up3 **(A)**, Uu10 **(B)**, Mh strains PG21 **(C)**, UAB 55391 **(D),** and Mpn strain M129 **(E)** in the presence of Neo with or without Cu supplementation. Graphs depict group mean ± SD (*n* = 3 replicates). Experiments were repeated 2–3 times at minimum.

To determine whether compounds with Cu-chelating ability may have antimicrobial activity against human mollicutes, MICs were performed with mollicutes for compounds with and without Cu supplementation. First, we examined the antimicrobial activity of Neo against human mollicutes. Neo activity has previously been examined on a closely related avian mycoplasma, *M. gallisepticum*, and its antimicrobial activity was shown to be Cu-dependent (Smit et al., 1982; Gaisser et al., 1987a,b; de Zwart et al., 1991). The ureaplasma strains showed differential susceptibility to Neo *in vitro* (Table 2). Up3 showed no growth inhibition at 10 μM, but had a >30-fold inhibition in the presence of exogenous Cu^2+^ when compared to 10 μM Neo alone (Figure 2A). Similarly, Uu9 also exhibited a >30-fold inhibition of growth in the presence of Neo and Cu^2+^ when compared to 10 μM Neo alone (Table 2). Uu10 had a twofold difference in growth inhibition, but this minimal inhibition may be due to a general increased Cu^2+^ sensitivity (Figure 2B). Screening of Neo on *M. hominis* strain PG21 showed an MIC of 1.25 μM with Neo and Cu^2+^ compared to 10 μM with Neo alone (an eightfold difference), and Mh strain 55391 had a MIC of 625 μM with Neo and Cu compared to Neo alone 10 μM, a 16-fold difference (Figures 2C,D and Table 2). The MIC for all four strains of Mpn with Neo and 50 μM Cu^2+^ was 1.25 μM while the MIC with Neo alone was 10 μM, an eightfold difference (Figure 2E and Table 2). As concentrations of >10 μM were not tested, toxicity of Neo alone on mollicutes’ growth was not determined as part of these studies.

**TABLE 2:** MIC ranges for human mollicutes with Neocuproine *in vitro*.

**Bacterial species**	**Strain**	**MIC with Neo**	**MIC with Neo + Cu**
*Ureaplasma parvum*	Up3	>10 μM	625 nM
*Ureaplasma urealyticum*	Uu9	>10 μM	625 nM
	Uu10	10 μM	5 μM
*Mycoplasma hominis*	PG21	>10 μM	1.25 μM
	UAB 55391	>10 μM	625 nM
	M129	10 μM	1.25 μM
*Mycoplasma pneumoniae*	FH	10 μM	1.25 μM
	UAB PO1	10 μM	1.25 μM
	UAB JZY	10 μM	1.25 μM

To exclude the potential role of other transition metals in the apparent Cu^2+^-specific antimicrobial effects of Neo, additional transition metals were tested. Mpn strains were cultured with Neo in the presence or absence of Cu^2+^, Fe^2+^, Mn^2+^, and Zn^2+^. As was seen previously, a 16-fold difference was present between Neo with Cu^2+^ compared to the Neo controls without added Cu^2+^ (Supplementary Figure 1). However, growth of Mpn with Neo in media supplemented with Fe^2+^, Mn^2+^, or Zn^2+^ was not significantly different from the control cultures despite the ability of Neo to form complexes with these metals (Xiao et al., 2011). These data suggest that Neo mediated antimicrobial activity is copper-dependent and independent of other transition metals (Supplementary Figure 1). All strains examined showed MICs of Neo with copper at about 1.25 μM while the MICs of Neo alone or Neo with the other transition metals were about 10 μM (data not shown). These data indicate that the enhanced inhibitory effect of Neo on Mpn growth is Cu^2+^ specific.

### GTSM and DSF Show Cu-Specific Antimicrobial Effects on *Mycoplasma* and *Ureaplasma* spp.

**FIGURE 3:**
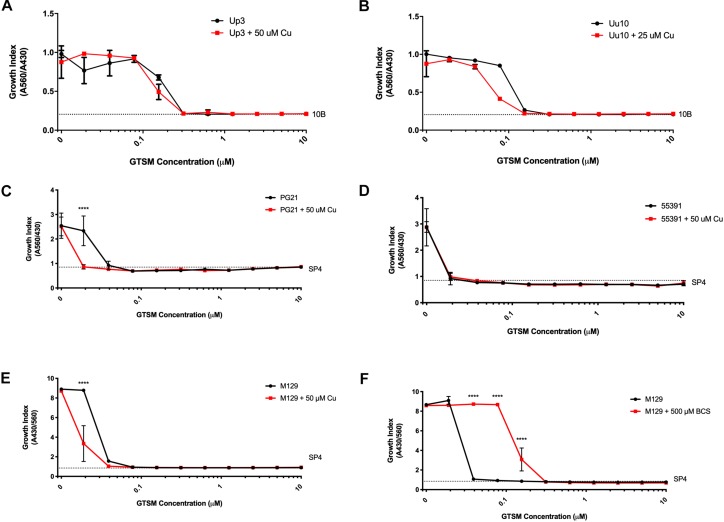
Glyoxal bis(4-methyl-3-thiosemicarbazone) shows Cu-enhanced toxicity *in vitro* against *Ureaplasma* and *Mycoplasma* species. Representative growth indices in the presence of GTSM with or without Cu supplementation of Up3 **(A)** and Uu10 **(B)**, Mh strains PG21 **(C)**, UAB 55391 **(D)**, and Mpn strain M129 **(E)**. Growth index of Mpn strain M129 in the presence of GTSM with or without bathocuproine **(F)**. Graphs depict group mean ± SD (*n* = 3 replicates). Experiments were repeated 2–3 times at minimum.

Glyoxal bis(4-methyl-3-thiosemicarbazone) is a compound previously shown to have copper-dependent antimicrobial activity against *M. tuberculosis* (Speer et al., 2013; Neyrolles et al., 2015). With respect to the ureaplasmas, Uu9 and Up3 showed growth inhibition at 313 nM GTSM, while strain Uu10 showed growth inhibition at 156 nM (Figures 3A,B and Table 3). This inhibition appeared to be independent of exogenous Cu supplementation. Interestingly, there was potent inhibition of Mh strains PG21 and 55391 with GTSM with an MIC of 40 and 20 nM, respectively, without Cu supplementation (Figures 3C,D and Table 3). Only PG21 showed a twofold difference with GTSM MICs, decreasing the MIC to 20 nM. All Mpn strains tested with GTSM showed inhibition of bacterial growth with a MIC of 40 nM. Growth inhibition did not appear to be altered with Cu^2+^ supplementation, suggesting that exogenous Cu^2+^ may not be necessary for GTSM to exhibit antibacterial activity in highly complex media (Figure 3E and Table 3).

**TABLE 3:** MICs for human mollicutes with GTSM *in vitro*.

**Bacterial species**	**Strain**	**MIC without Cu (nM)**	**MIC with Cu (nM)**
*Ureaplasma parvum*	Up3	313	313
*Ureaplasma*	Uu9	313	313
*urealyticum*	Uu10	156	156
*Mycoplasma*	PG21	40	20
*hominis*	55391	20	20
	M129	40	40
*Mycoplasma*	FH	40	40
*pneumoniae*	UAB PO1	40	40
	UAB JZY	40	40

To determine whether GTSM inhibited mycoplasma and ureaplasma growth at least partly by forming complexes with the low level of Cu^2+^ in the growth media, BCS, a membrane impermeable copper-binding compound, was utilized to sequester free Cu^2+^ ions in the growth medium. MICs for GTSM were carried out on Mpn in the presence or absence of BCS, without exogenous Cu^2+^ supplementation. GTSM activity was decreased in the presence of BCS, altering MICs from 40 to 156 nM (Figure 3F and Table 4) against all Mpn strains examined. These increased MICs suggest that the trace amounts of Cu^2+^ in standard medium are sufficient to boost the growth inhibitory activity of GTSM, and also suggests that GTSM may also act in a copper-independent manner at higher concentrations.

**TABLE 4:** MICs for Mpn with GTSM ± BCS *in vitro*.

**Bacterial species**	**Strain**	**MIC with GTSM (nM)**	**MIC with GTSM + BCS (nM)**
	M129	40	156
*Mycoplasma*	FH	40	156
*pneumoniae*	UAB PO1	40	156
	UAB JZY	40	156

**FIGURE 4:**
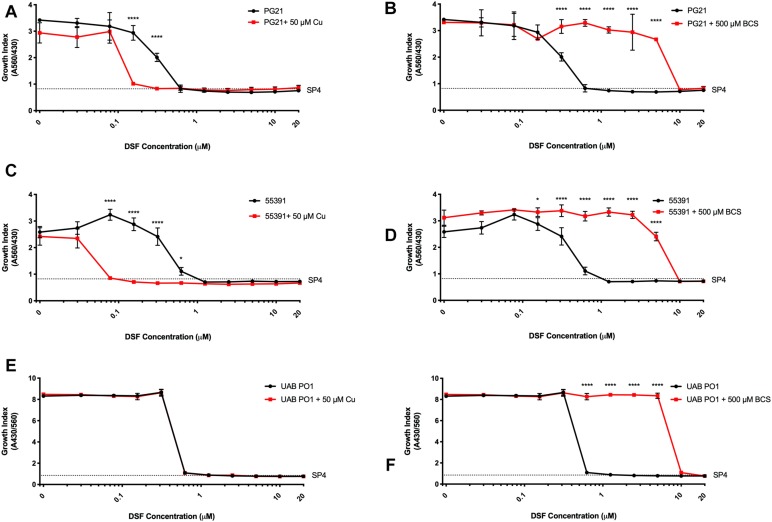
Disulfiram shows toxicity against *Mycoplasma* species *in vitro*. Representative growth indexes of *M. hominis* PG21 **(A)** in the presence of DSF, with or without Cu supplementation (50 μM), and BCS resue (500 μM) **(B)**. Representative growth indexes of UAB 55391 **(C)** in the presence of DSF, with and without Cu supplementation (50 μM), and BCS resume (500 μM) **(D)**. Representative growth index of Mpn strain M129 **(E)** in the presence of DSF with or without Cu (50 μM), and BCS rescue (500 μM) **(F)**. Graphs depict group mean ± SD (*n* = 3 replicates). Experiments were repeated 2–3 times at minimum.

Having shown that membrane-permeable copper-binding compounds can exhibit growth inhibitory activity against human mollicutes, we examined the FDA-approved drug DSF for similar activity, as it has been shown previously to have copper-dependent antimicrobial activity against other bacterial species (Dalecki et al., 2015). DSF was examined for growth inhibitory activity against the Mh type strain PG21 and a MDR clinical isolate (55391) with or without exogenous Cu supplementation. Both strains showed MICs at 625 nM DSF without Cu^2+^ addition to media (Figures 4A,C). With addition of 50 μM Cu^2+^ in the presence of DSF, the MIC for PG21 to DSF showed a fourfold decrease to 156 nM (Figure 4A and Table 5). Strain 55391 showed an eightfold difference in DSF susceptibility with Cu^2+^ addition, with a MIC of approximately 78 nM (Figure 4C).

**TABLE 5:** MICs for *Mycoplasma* spp. with DSF ± exogenous Cu alone or Cu and BCS.

**Bacterial species**	**Strain**	**MIC with DSF (nM)**	**MIC with DSF + Cu (nM)**	**MIC with BCS (μM)**
*Mycoplasma*	PG21	625	156	10
*hominis*	55391	625	78	10
	M129	625	625	10
*Mycoplasma*	FH	625	625	10
*pneumoniae*	UAB PO1	625	625	10
	UAB JZY	625	625	10

To show that DSF was utilizing Cu^2+^ to increase MICs in Mh strains, BCS was utilized without exogenous Cu^2+^ supplementation. Upon addition of BCS to DSF-containing cultures, MIC values for both PG21 and 55391 shifted from 625 nM to 10 μM DSF (Table 5 and Figures 4B,D). DSF, in addition to intermediate μM MIC values against Mh, showed MIC values of 0.625 μM against Mpn strains tested (Figure 4E and Table 5), which increased to 10 μM with addition of BCS (Figure 4F). These data suggest that DSF, an FDA-approved, copper-binding drug, exhibits copper-dependent antimicrobial activity against Mh.

## Discussion

Nutritional immunity is a relatively new field which has arisen upon observations of utilization of specific transition metals by phagocytes against infective microbes. While metal starvation is one scheme for immunologic sequestration of nutrients required for bacteria growth (Fe^2+^ and Mn^2+^), use of metals such as copper in the phagolysosome has been described as one mechanism of cellular antimicrobial activity (Didier et al., 2010; Samanovic et al., 2012; Parrow et al., 2013; Neyrolles et al., 2015). During the process of phagolysosome destruction of infectious microbes, ATP7A, a copper-transporting ATPase, pumps Cu^1+^ ions into the phagolysosome, thereby creating an environment saturated with transition metals. In conjunction with other host defenses, this contributes to eventual destruction and clearance of microbes *in vivo* (White et al., 2009; Ladomersky et al., 2017). Thus, Cu^1+^ ions are important during microbial killing by eukaryotic cells, and not unexpectedly resistance mechanisms also exist that permit bacterial endurance of transition metal exposure, demonstrating the co-evolution of traits for host defense and pathogenesis. P-Type ATPases exist in bacterial species, alongside a large number of transporters, chaperone proteins, and myriad means of Cu chelation (Rensing and Grass, 2003; Shafeeq et al., 2011; Wolschendorf et al., 2011; Johnson et al., 2015). Of the many documented copper resistance mechanisms in related prokaryotic species currently the only annotated genes present in human mollicutes are P-type ATPases (i.e., *MgtA*) (Alex et al., 2017). Further work will be required to identify the genes required for resistance by different species of mollicutes to copper-mediated toxicity.

Little work has been carried out with respect to antimicrobial effects of transition metals on human mollicutes. Previously published work with *S. aureus* and *M. tuberculosis* has demonstrated that compounds that chelate copper can exhibit antibacterial effects (Dalecki et al., 2016; Shah et al., 2016). Previous work in another laboratory during the 1980s examined the effect of Neo on Cu stress with *M. gallisepticum* (Smit et al., 1982; Gaisser et al., 1987b). The mechanism of action for Neo was determined to be the result of copper toxicity, and not that of the ligand itself (Smit et al., 1982). This was also found for DSF and its activity against *M. tuberculosis* where the drug was described as a Trojan horse, disguising copper during transition into the bacterial cell and thus preventing exclusion (Dalecki et al., 2015). These data showed potential benefit for drug discovery against an avian pathogen. Sensitivity to transition metals, namely Mn^2+^, has been reported in *U. urealyticum*, prior to the separation of *U. parvum* from the same species (biovar) classification (Robertson and Chen, 1984). Interestingly, all Up isolates (serovars 1, 3, 6, and 14) had a transient growth inhibition with Mn addition, but this was permanent for Uu isolates (serovars 2, 4, 5, 7, 8, 9, 10, 11, and 12) and could not be rescued with the addition of Cu. One probable explanation for the failure of Cu^2+^ to rescue Mn^2+^-mediated growth inhibition on *Ureaplasma* spp. is the relatively low MIC for Cu^2+^, which our work has explored. As compared to the other examined *Mollicutes* spp., Mpn was comparatively more resistant to copper-augmented stress with Neo and DSF. The increased resistance of Mpn to Cu^2+^ ion stress compared to the examined spp., in general, may explain these differences, although more extensive work should be carried out to determine if this is true for additional mycoplasma and *Ureaplasma* spp.

This is the first report to detail the toxicity of copper against human mycoplasmas and ureaplasmas as well as documenting copper-dependent antimicrobial activity of three different copper-binding compounds. We found a high Cu^2+^ MIC for isolates of the lung pathogen Mpn and an increased Cu^2+^ sensitivity in the urogenital organisms Mh and *Ureaplasma* isolates. The antimicrobial effects of copper seen with the type strains of Mpn, Mh, Uu and Up with the compounds tested in this study were similar with the drug-resistant isolates Uu serovar 9 (*tetM* positive), and Mh 55391 (resistant to macrolides, tetracyclines, and fluoroquinolones). We speculate that this difference may be due in part to their “normal” anatomic niche, whereby respiratory pathogens must be more resistant to Cu^2+^ due to increased numbers of macrophages in the respiratory tract. Other respiratory bacteria (i.e., *M. tuberculosis* and *Streptococcus pneumoniae*) have been reported have multiple resistance mechanisms to Cu^2+^, and loss of these response factors during acute phagocyte-derived copper exposure can decrease both virulence and longevity of infections (Shafeeq et al., 2011; Wolschendorf et al., 2011; Johnson et al., 2015). However, contrary to this hypothesis of a role for anatomic niche in copper resistance, the *cop* operon, such as that found in *S. pneumoniae*, is present in urogenital pathogens such as uropathogenic *Escherichia coli* (Rensing and Grass, 2003). Additionally, copper can be found in urine as a host effector molecule to decrease bacterial infiltration into the urinary tract (Hyre et al., 2017). Relatedly, mollicutes typically found in the urogenital tract have also been reported in oropharyngeal and respiratory specimens (Waites et al., 2005; Bharat et al., 2015; Tyner et al., 2016; Atkinson et al., 2018). These conflicting findings suggest that the reason(s) for these observed differences in copper-resistance will require further study.

There are some limitations in our study. SP4 and 10B may be some of the richest microbial culture media currently used, and contain ∼15% serum (Waites et al., 2012a). Thus, our testing was carried out using media containing poorly defined biologic components (e.g., serum, peptone, tryptone, and yeast extract), increasing the potential for finding false positives and false negatives on copper-dependent drug compounds. We did, however, measure the total copper levels in the media and found them to be much lower than the concentrations chosen for testing. This study furthermore does not attempt to determine whether mechanisms of resistance could be uncovered with prolonged exposure of these compounds *in vitro*. Due to a lack of reliable metrics for consistent MIC analysis of *Mycoplasma genitalium*, and lack of recent clinical isolates, this organism was excluded from these analyses. This organism may be sufficient to explain the hypotheses pertaining to anatomical site-specific Cu^2+^ resistance, but further work is required. Finally, these observations are entirely limited in scope to *in vitro* MIC work, and extensive testing *in vivo* will be required before usage in patients is possible.

In conclusion, this study has demonstrated differing ranges of Cu^2+^ toxicity between Mpn, Mh, Uu, and Up. Neo, GTSM, and DSF exhibit interesting, copper-dependent antimicrobial activity against mycoplasmas and ureaplasmas that merits further investigation.

## Data Availability

The raw data supporting the conclusions of this manuscript will be made available by the authors, without undue reservation, to any qualified researcher.

## Author Contributions

AT, LX, FW, and TA contributed to the design and implementation of the study. AT, CC, and AD performed the experimental studies and analysis. All authors aided in interpretation and outcomes of experimental analyses, contributed to the revisions and editing of this manuscript, and read and approved the final version of the manuscript for submission. AT and TA wrote the first draft of the manuscript. CC, AD, LX, and FW extensively edited the subsequent drafts of the manuscript.

## Conflict of Interest Statement

The authors declare that the research was conducted in the absence of any commercial or financial relationships that could be construed as a potential conflict of interest.

## Abbreviations

BCS: bathocuproine disulfonate
CuSO_4_: copper sulfate
DSF: disulfiram [bis(diethylthiocarbamoyl) disulfide]
FDA: US Food and Drug Administration
GTSM: glyoxal bis(4-methyl-3-thiosemicarbazone)
ICP-MS: inductively coupled plasma mass spectrometry
MDR: multidrug resistant
Mh: *Mycoplasma hominis*
Mpn: *Mycoplasma pneumoniae*
Neo: neocuproine (2,9-dimethyl-1,10-phenanthroline)
Up3: *Ureaplasma parvum* serovar 3
Uu9/Uu10: *Ureaplasma urealyticum* serovars 9/10.
