# Supplementary material for: Differential Susceptibility of Mycoplasma and Ureaplasma Species to Compound-Enhanced Copper Toxicity
Source: Front Microbiol. 2019 Jul 30;10:1720. doi: 10.3389/fmicb.2019.01720 (PMC6682632; doi:10.3389/fmicb.2019.01720)
Supplement: Supplementary file 1 [file Data_Sheet_1.docx]

Supplementary Material


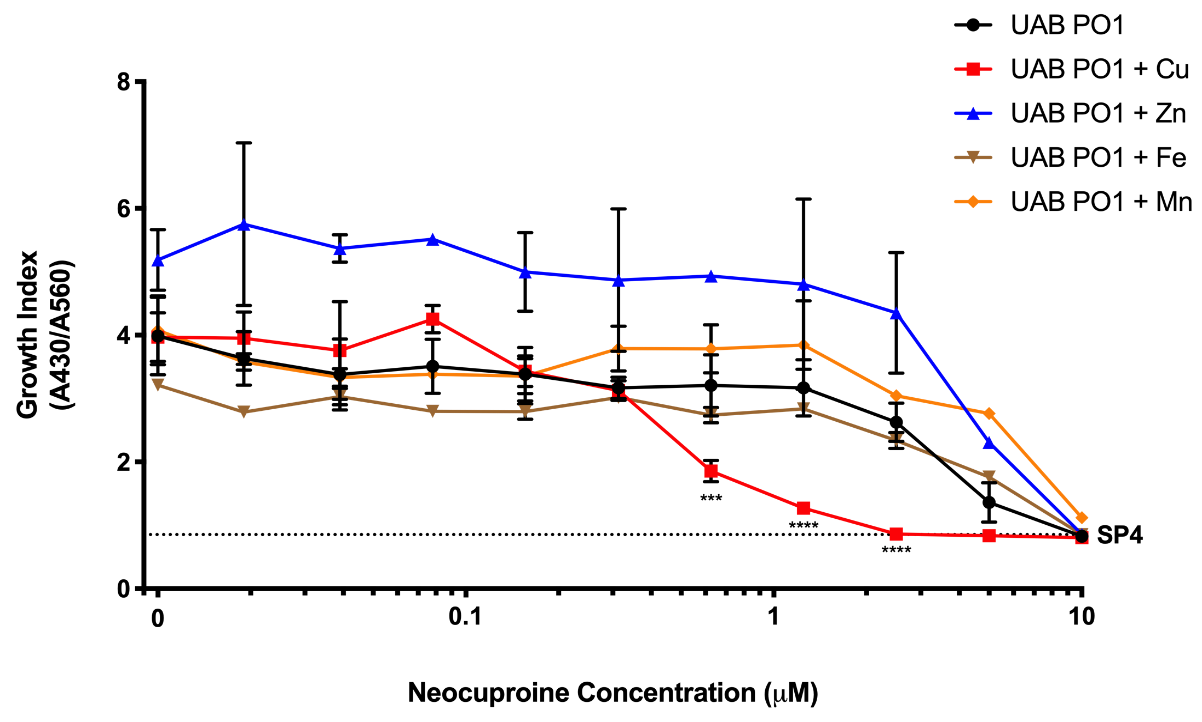


**Supplementary Figure 1.** **Neocuproine shows Cu-selective toxicity *in vitro* against *Mycoplasma pneumoniae*, compared to other transition metals.**  Representative growth index of Mpn strain UAB PO1 in the presence of Neocuproine with Cu, Zn, Fe and Mn (all metals at 50 μM). Graph depicts group mean ± Std Deviation (n = 3 replicates). Experiments were repeated 2-3 times at minimum.
